# *In-vitro* antiviral activity of *Carica papaya* formulations against dengue virus type 2 and chikungunya virus

**DOI:** 10.1016/j.heliyon.2022.e11879

**Published:** 2022-11-30

**Authors:** P. Patil, K. Alagarasu, D. Chowdhury, M. Kakade, S. Cherian, S. Kaushik, J.P. Yadav, S. Kaushik, D. Parashar

**Affiliations:** a ICMR-National Institute of Virology, 20-A, Dr. Ambedkar Road, Pune 411001, Maharashtra, India; b Maharshi Dayanand University, Rohtak, Haryana, India

**Keywords:** Antiviral, *Carica papaya*, Dengue virus, Chikungunya virus

## Abstract

Dengue and chikungunya are diseases of global health significance and currently, no antivirals are available to treat these arboviral diseases. *Carica papaya* leaves extract is traditionally used to treat thrombocytopenia in patients infected with the dengue virus. The current study was undertaken to study the antiviral activity of commercially available *Carica papaya* leaves extract (CPLE) based products and CPLE prepared in four formulations against dengue virus type 2 (DENV-2) and chikungunya virus (CHIKV). Maximum nontoxic concentrations of the commercially available CPLE based products and CPLE based formulations (*Carica papaya* leaves in powder form, *Carica papaya* leaves in lyophilized form, *Carica papaya* leaves based silver nanoparticles and supercritical fluid extract of *Carica papaya* leaves) were used for screening the antiviral activity. The antiviral activity against DENV-2 and CHIKV were assessed post infection using focus forming unit assay. Effective formulations were tested under different conditions i.e. pretreatment, cotreatment and posttreatment. The virus output after treatment was assessed by real-time RT-PCR, immunofluorescence assay and focus forming unit assay. The results revealed *Carica papaya* leaves based silver nanoparticles and supercritical fluid extract of *Carica papaya* leaves formulations showed significant inhibition in case of DENV while papaya leaves in powder form showed significant reduction in case of CHIKV. This study demonstrates the antiviral activity of CPLE formulations against DENV-2 and CHIKV infection in *in-vitro* system and needs further validation in *in-vivo* models.

## 1 Introduction

Dengue (DEN) and Chikungunya (CHIK) viruses and their co-infections have emerged as major public health threats in tropical and sub-tropical regions. With the lack of licensed vaccines and antivirals, natural compounds present an exciting possibility to explore because of their expected low side effects, high accessibility in nature and cost effectiveness for low income countries [1]. There have been several studies on the antiviral activity of various phytochemicals against DENV and CHIKV including various flavonoids, polysaccharides, ethers, alkaloids, terpenoids, terpenes, essential oils etc. [2]. Studies have shown that extracts from different parts of medicinal plants provide better anti-DENV/CHIKV activity as compared to their synthetic analogues [3, 4, 5, 6, 7, 8, 9, 10, 11, 12].

Over the centuries, papaya (family Caricaceae) has been explored for its nutraceutical and cosmeceutical properties in Africa, Asia, and Europe. Traditionally, it is a renowned traditional medicine for many ailments including viral diseases. Scientific studies have demonstrated the use of papaya for its anti-bacterial, anti-inflammatory, nephroprotective, antiparasitic, antidiabetic, hepatoprotective, and anti-dengue properties [13]. Although comprehensive investigation on the bioactive compounds is needed to understand the mechanism of action, positive conclusions are reported from various studies to validate the use of papaya leaves for the dengue fever treatment and have shown effectiveness in refining thrombocytopenia using *in-vitro* and *in-vivo* systems [14]. Multiple small-scale studies and case reports suggest a rapid increase of platelet count in the patients after management of papaya leaf extract and attribute its erythrocyte membrane-stabilizing potential as a possible reason for this effect [14, 15]. Direct antiviral activity has been demonstrated in the *in-vitro* studies wherein THP-1 cells treated with papaya leaves extract (PLE) showed a significant decrease in DENV envelope and NS1 protein expression, decreased viral load, and an increase in the expression of Type I interferons (IFN-α) [10]. *In-vivo* study using DENV infected mice showed modulation of genes associated with endothelial permeability regulation in the liver after treatment with papaya leaf extract. Although the mechanism of action is not clear, studies have reported the anti-DENV activity of *Carica papaya* extracts on the DENV infected cells with inconsistent results [16, 17, 18]. The methods used to assess antiviral activity in these studies are not robust. There is limited scientific literature available for studies showing antiviral activity against CHIKV using *Carica papaya*. The solvents used for extraction might affect the composition of active components. Moreover, nanoparticle based formulations provide for targeted medication administration, improved bioavailability, and the maintenance of drug or gene effects in target tissues, as well as improved stability. In the fields of nanotechnology and nanomedicine, silver nanoparticles have played a vital role. It was hypothesized that the formulations might vary in their composition of active compounds and differ with regard to their antiviral activity. Hence the objective of the present study was to test four papaya formulations i.e. papaya leaves in powder form (PP), papaya leaves in lyophilized form (PL), *Carica papaya* leaves based silver nanoparticles (PNP) and supercritical fluid (SF) extract of *Carica papaya* leaves (PE) and commercially available drugs containing papaya as an active ingredient for the *in-vitro* antiviral activity against DENV-2 and CHIKV. The antiviral activity was investigated using focus forming unit (FFU) assay, quantitative RT-PCR (qRT-PCR) and immunofluorescence (IFA) assay.

## 2 Materials and methods

### 2.1 Plant material collection

Fresh, healthy, and well-grown leaves of *Carica papaya* were taken from the herbal garden of Maharshi Dayanand University, Rohtak, Haryana, India. The geographical location of the collection site of the plants is Latitude 280 53′24.97″N, Longitude-760, 34′57.252″E. The *Carica papaya* plant specimen was recognized and certified by comparing it to the herbarium at the Forest Research Institute (FRI), Dehradun, India with the Voucher No. D. DUN 25636. The same specimen was deposited with Voucher No. MDU 3201 in the Department of Genetics, Maharshi Dayanand University, Rohtak, Haryana, India. All methods were performed in accordance with the relevant guidelines and regulations.

### 2.2 Preparation of different formulations

#### 2.2.1 *Carica papaya* leaves in powder form (PP)

Fresh, healthy, and well-grown leaves of *Carica papaya* were washed, air-dried at room temperature in shaded area for one week and pulverized to form a powder.

#### 2.2.2 *Carica papaya* leaves in lyophilized form (PL)

*Carica papaya* leaves were meticulously washed, air-dried at room temperature in shaded area for a week's time and ground. These dry and pulverized leaves were then heat treated for 25 min in sterilized double-distilled water, allowed to cool, filtered and lyophilized.

#### 2.2.3 *Carica papaya* leaves based silver nanoparticles (PNP)

Leaves of *Carica papaya* were added with sterile double-distilled water and heated for 25 min. Green AgNPs of *Carica papaya* were standardized by adding various concentrations of the aqueous leaves extract in 1mM silver nitrate solution (169.87 mg/L). The *Carica papaya* extract was treated with AgNO_3_ aqueous solution and was incubated at room temperature for 24 h. The solution changed from yellow to yellowish-brown colour within 10–15 min of mixing indicating the reduction of AgNO_3_ to Ag+ ions. After incubation, the solution was centrifuged and washed thrice with double distilled water to remove unbounded capping materials. The final solution was lyophilized for further use. The size and stability of nanoparticles were assessed by dynamic light scattering (DLS) and Zeta potential before using DLS-Malvern Zetasizer NanoZS (Malvern Panalytical, UK).

#### 2.2.4 Supercritical fluid extract of *Carica papaya* (PE)

SF extraction extract of *Carica papaya* plant was prepared using SF extraction machine (Speed™ SFE Prime of Applied Separation Inc. U.S.). Every time, ten grams of *Carica papaya* fine plant powder was loaded into the machine chamber. The solvent (CO_2_) flow rates were changed from 1.6 ml/min in static dynamic mode (1 h static and 30 min dynamic mode). Different pressure parameters were tuned for the separation of secondary metabolites.

### 2.3 Preparation of papaya extracts stocks

All the papaya leaves-based formulations were dissolved in 50% each of DMSO and PBS to make stocks at the concentration of 10 mg/ml. The stocks were sterile filtered using 0.22 μm filters and stored at −20 °C for further use.

### 2.4 Preparation of stocks for commercial drugs

Commercially available drugs containing papaya as an active ingredient i.e. Papayen (Bio Dharampur Farms), Tcyte (McW healthcare Pvt. Ltd), Platenza (Himalaya Healthcare), Platex Forte (Zoic Pharmaceuticals) and Reditus (Sanchomee Herboveda Pvt. Ltd.) were dissolved in MEM to obtain 500 μg/ml stock solution (Supplementary Table 1).

### 2.5 Cell culture and virus stock

Vero (ATCC No. CCL-81) cells was maintained using MEM (Himedia), supplemented with 10% FBS (Gibco, US Origin) and Antibiotic-Antimycotic (Sigma-Aldrich) at 37°C and 5% CO_2_. Dengue virus (DENV) Serotype-2 (Strain no. 803347) and Chikungunya virus (CHIKV, Strain no. 061573) were used for this study. DENV-2 strain was used in this study since it is the most prevalent serotype in India [19]. DENV-2 strain stock was prepared in C6/36 and CHIKV stock was propagated in Vero cells, and stored at −80°C.

### 2.6 Cytotoxicity and cytopathic effect inhibition assay

Cytotoxic effect of papaya formulations (commercial and plant material formulations) on Vero CCL-81 cells were evaluated using 3-(4,5-dimethythiazol-2-yl)-2,5-diphenyl tetrazolium bromide (MTT) assay [23]. Briefly, Vero cells were incubated with different concentrations of test formulations (0–200 μg/ml) at 37°C for 5 days. After that, cells were incubated with MTT solution (5 mg/ml) at 37°C for 3 h. The solubilized formazan crystals were measured using a microplate reader (BioTek Synergy, USA) at 570 nm. The percent inhibition and CC-50 values were calculated.

### 2.7 Screening of formulations for inhibitory effects against DENV-2 and CHIKV

The formulations were screened for antiviral activity under post infection condition against DENV-2 and CHIKV. The maximum nontoxic dose (MNTD) of each formulation was used for screening. The confluent monolayers of cells in 24 well plates were infected with 0.1 multiplicity of infection (MOI) DENV-2 and 0.01 MOI for CHIKV for 1 h and treated with the formulation after infection. The cells infected with DENV-2 were incubated for 5 days and those infected with CHIKV were incubated for two days post infection. In the virus control, wells contained only the infected cells without any formulations while the cell control contained uninfected cells only. After incubation, the culture supernatants were collected after freezing the plates at −80°C followed by thawing. The collected supernatant was used for the determination of titre of infectious virus particles by focus forming unit (FFU) assay.

### 2.8 Antiviral activity of formulations in *in-vitro* system

The formulations which show the statistically significant reduction of virus titre in comparison to the virus control (wells without test formulations) in the screening process were further studied under pretreatment, cotreatment, and posttreatment conditions with different nontoxic concentrations of the drugs.

During pretreatment, the cells were first treated with formulations for 24 h and kept at 37°C. After that culture supernatant was discarded and the cells were infected with DENV-2or CHIKV and kept at 37°C for 1 h. Unbound virus particles were removed by PBS wash twice and incubated after the addition of maintenance media with antibiotics and 2% FBS.

In cotreatment, the virus was treated with various concentrations of the formulation and the cells were infected with virus and formulation mixture for 1 h. In post treatment, the cells were infected with 0.1 MOI DENV-2 or 0.01 MOI for CHIKV for 1 h and treated with the formulations. For all the treatments, the plates were incubated for five and two days in case of DENV-2 and CHIKV respectively. After incubation, the plates were freezed at −80°C and thawed for collection of culture supernatant. Then supernatant was used for estimation of virus titre by FFU assay. All the experiments were done in triplicates.

### 2.9 Quantitative RT PCR, Focus forming unit assay and immunofluorescence assay

Magnetic beads based technology was used to isolate RNA using commercial extraction kits (MagMAX-96 Viral RNA Isolation Kit, Thermo Fisher Scientific). RNA detection and quantitation was done using qRT-PCR as described earlier [20, 21, 22]. Briefly, the RNA was extracted and added to a mixture containing DENV-2 or CHIKV specific primers and probes described earlier [20, 21, 22], and amplified using superscript III One step RT-PCR system containing platinum Taq DNA polymerase. FFU assays for DENV-2 and CHIKV were performed as described earlier to find out the titre of viruses [[21], [22]]. IFA assay was performed for the estimation of virus infectivity as described earlier [20, 21, 22].

### 2.10 Statistical analysis

The log titres of viral RNA/FFU or percent infected cells were compared between the different groups using one ANOVA followed post correction tests. A P value of less than 0.05 was considered significant. The statistical analysis was done using GraphPad prism software version 7.0.

## 3 Results

### 3.1 *Carica papaya* leaves based formulations exert anti-dengue and anti-chikungunya activity

#### 3.1.1 PNP and PE based formulations exert antiviral activity against DENV

A change in color of the solution from pale green to light brown confirmed the synthesis of nanoparticles. The nano-silvers in the colloidal solution was confirmed by the results of spectrum scan which yielded a sharp peak at 420 nm. The average size of nanoparticles in the colloidal system was 76.27 nm. The average Zeta potential of *Carica papaya* leaf silver nanoparticles was −17.4 mV.

**Figure 1:**
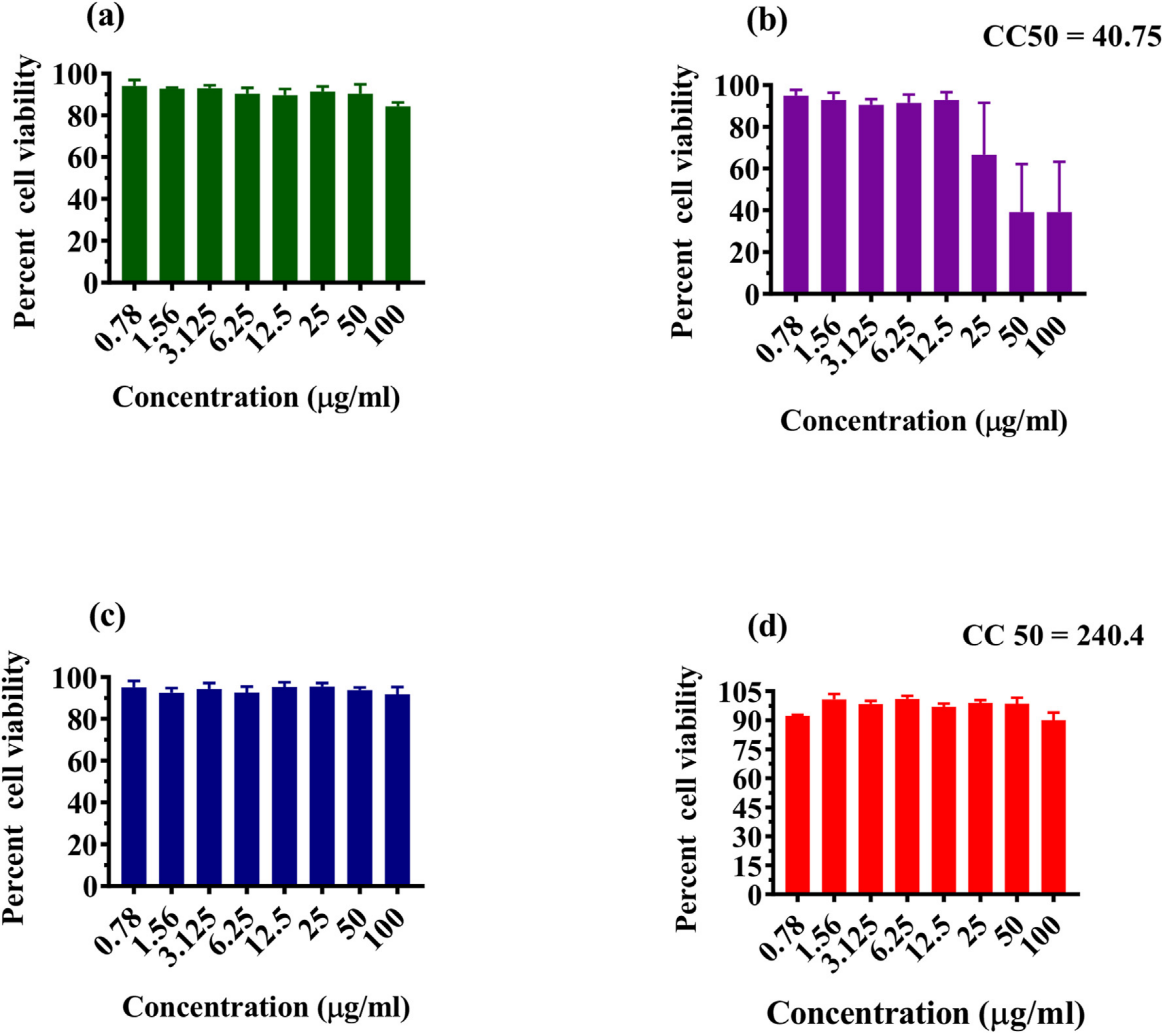
Cell viability of papaya based formulations at various concentrations. Vero CCL-81 cells were cultured with different concentrations of formulations i.e. PP (a), PL (b), PNP (c), PE (d) for 5 days at 37 °C, treated with MTT solution for an another 3 h at 37 °C. After incubation, the medium was aspired and acidified isopropanol was added to the wells and kept at 37 °C for 1 h and readings were measured using a spectrophotometer. Percentage inhibition and CC-50 values were calculated in comparison with cells untreated with formulations. CC-50 values were calculated based on non-linear regression analysis using GraphPad prism software version 7.0. The results were expressed as mean percent viability ± standard error of mean (SEM). All the experiments were performed in triplicates.

The optimum supercritical condition for *Carica papaya* formulation was determined to be 60°C at 200 bar pressure, yielding 0.25 g from 10 g of fine papaya powder. Potential cytotoxic effects of the four formulations i.e. *Carica papaya* leaves in powder form (PP), *Carica papaya* leaves in lyophilized form (PL), *Carica papaya* leaves extract based silver nanoparticles (PNP) and SF extract of *Carica papaya* leaves (PE), were determined using the MTT assays. PNP and PP showed no cytotoxicity against Vero CCL-81 cells. PE and PL showed CC50 values of 240.4 μg/ml and 40.75 μg/ml respectively (Figure 1). These four formulations were further evaluated for anti-dengue and anti-chikungunya activity.

**Figure 2:**
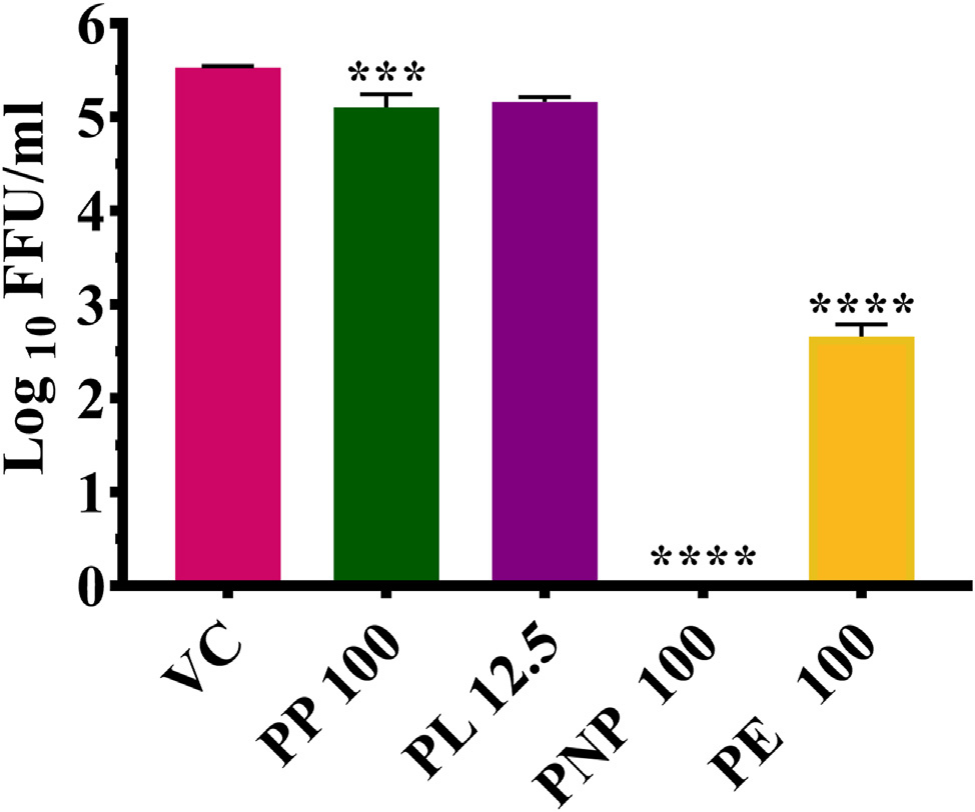
Primary screening of papaya formulations using FFU assay against DENV. DENV-2 infected Vero CCL-81 cells were treated with the formulations and were incubated for 5 days' post infection. After incubation, the plates were freeze thawed, and the culture supernatant was used for the determination of titre of infectious virus particles by FFU assay. The results were expressed as mean log10FFU± SEM. All the experiments were performed in triplicates. ∗∗∗∗p < 0.0001; ∗∗∗p < 0.001; ∗∗p < 0.01 and ∗p < 0.05 vs. virus control (VC).

Primary screening was performed to identify the potential DENV & CHIKV inhibitors under post infection treatment conditions. Out of four formulations, PNP exerted a 100% reduction (p < 0.0001) while PE showed significant reduction (5.505–3.20 mean log_10_ FFU/mL value) in DENV-2 titre compared to virus control (Figure 2).

**Figure 3:**
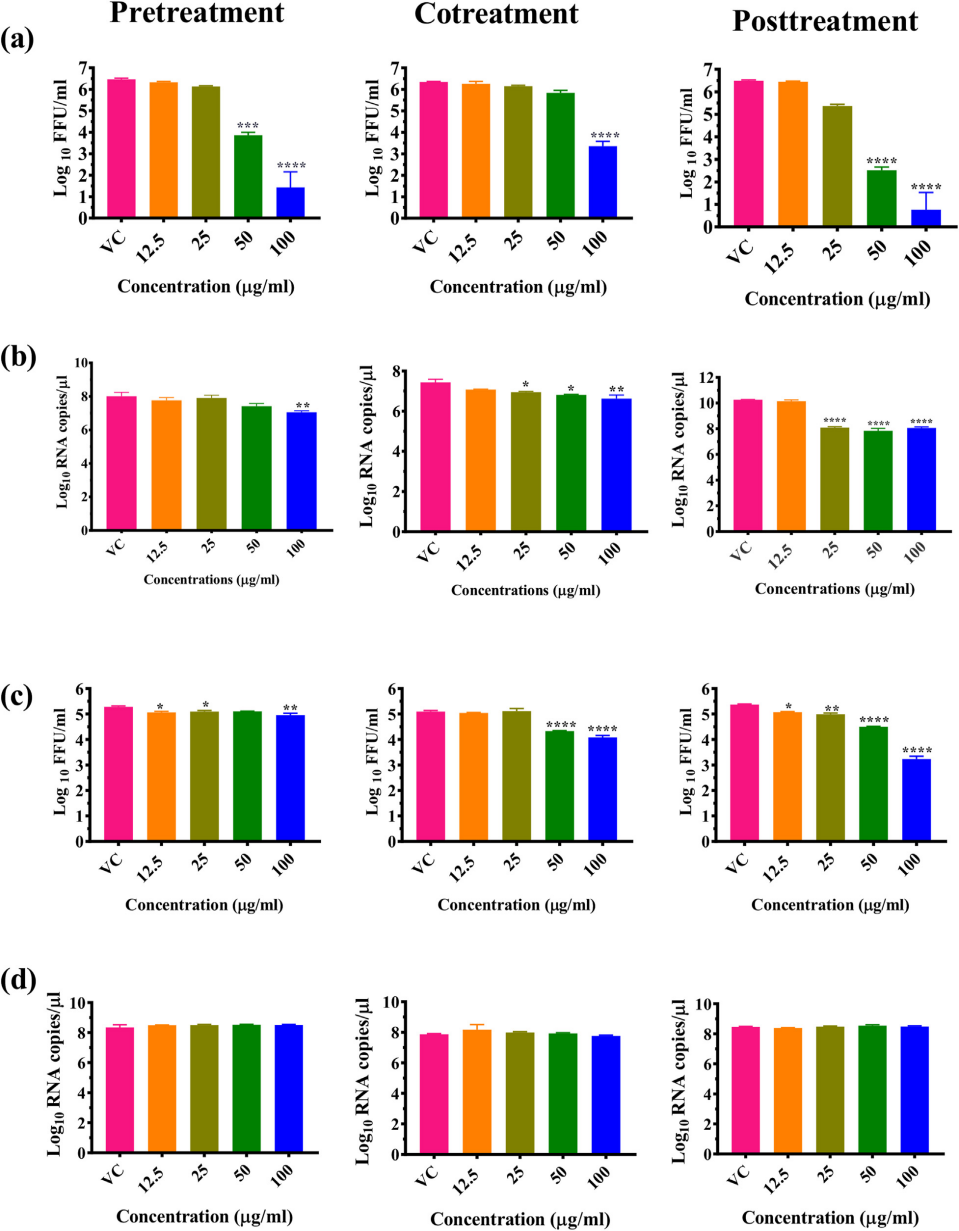
Effect of *Carica papaya* nanoparticles and papaya extract under pretreatment cotreatment and posttreatment conditions on DENV-2 by FFU assay (a,c) and qRT-PCR (b,d). Vero CCL-81 cells were treated with different concentrations (12.5, 25, 50 and 100 μg/ml) of PNP (a, b) and PE (c, d) under different conditions. After incubation, the plates were freezed and the culture filtrates were used for FFU and qRT-PCR assays. Mouse anti-dengue antibody was used as a primary antibody and goat anti-mouse IgG HRP conjugate was used as a secondary antibody in the FFU assay. For qRT-PCR, viral RNA was isolated and used for estimating viral RNA copies using real-time RT-PCR. The results were expressed as mean log_10_RNA copies/μl or log_10_FFU/ml± SEM. All the experiments were performed in triplicates at different time points. ∗∗∗∗p < 0.0001; ∗∗∗p < 0.001; ∗∗p < 0.01 and ∗p < 0.05 vs. virus control (VC).

Further investigation of PNP and PE for anti-dengue activity under pretreatment, cotreatment and post infection treatment conditions was done. Results showed a significant reduction of viral titer from 6.46 (VC) to 3.86 and 1.43 mean log_10_ FFU/mL at 50 μg/ml (p < 0.001) and 100 μg/ml (p < 0.0001) respectively in case of cells pretreated with PNP. In case of cotreatment, 100 μg/ml PNP showed significant reduction from 6.34 (VC) to 3.35 log_10_ FFU/mL (p < 0.0001). When the cells were treated with PNP after infection, ∼99% reduction of viral foci (p < 0.0001) was observed at 100 μg/ml from 6.48 (VC) to 0.76 log_10_ FFU/mL (p < 0.0001) and significant reduction at 50 μg/ml from 6.48 (VC) to 2.51 log_10_ FFU/mL (Figure 3a). PNP treatment resulted in a significant reduction in the log_10_ titer of viral RNA copy number under posttreatment condition from 25 μg/ml onwards (p < 0.0001) while a <1 log reduction in the DENV RNA copy number was observed under pretreatment and cotreatment conditions (Figure 3b).

Posttreatment of cells with 100 μg/ml concentration of PE reduced the DENV titre from 5.37 to 3.23 mean log_10_ FFU/ml (p < 0.0001). When the virus was mixed with PE and used for infection (cotreatment) ∼1 log reduction of the virus titer was noted at 100 μg/ml concentration (p < 0.0001). In the case of pretreatment, less than 1 log reduction in FFU titre was noticed (Figure 3c). For PE, quantitative RT-PCR showed a non-significant decrease in copy number of DENV RNA in all treatment conditions (Figure 3d).

**Figure 4:**
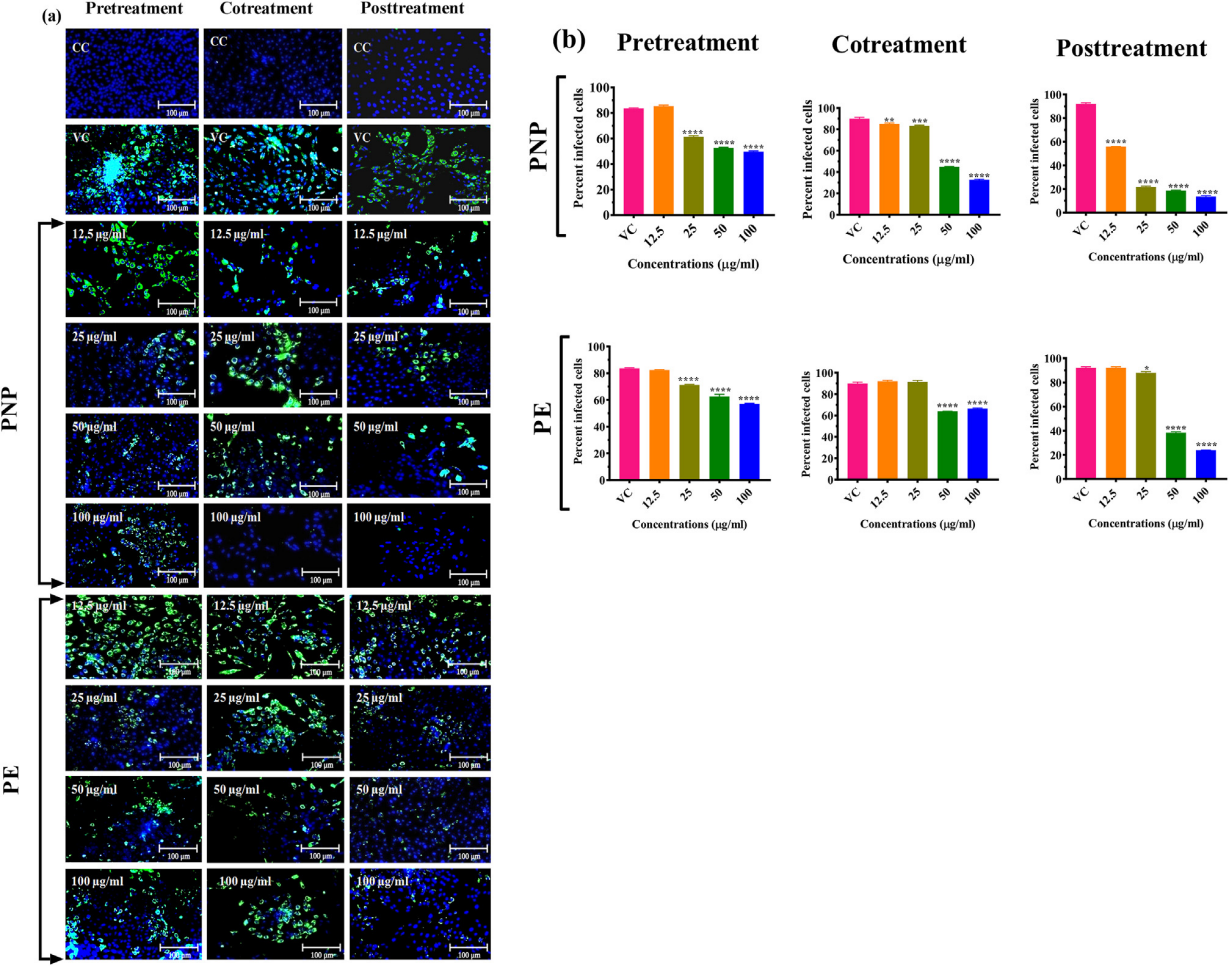
Effect of PNP and PE on DENV infection as measured by immunofluorescence assay. (a) Immunoflourescent images of Vero CCL-81 cells infected with DENV-2 and treated with various concentrations of PNP and PE under different treatment conditions. Green colour cells indicate virus infected cells. (b) Percentage of infected Vero cells in cultures treated with various concentrations of PN and PE under different treatments. Cells were counted in three different fields to obtain the per cent infected cells. The results were expressed as mean percent infected cells ± SEM. All the experiments were performed in triplicates. ∗∗∗P < 0.001, ∗∗P < 0.01 and ∗P < 0.05 vs. virus control (VC).

IFA was also performed to detect DENV-2 antigen in the infected cells and determine the extent of inhibition of infection by different effective papaya formulations at different concentrations. Results showed the dose dependent reduction of DENV-2 antigen in cells treated with PNP and PE (Figure 4a and b). The most significant reduction of virus infection was observed in cells treated with 100 μg/ml concentration of PNP and PE under post treatment conditions.

#### 3.1.2 PP based formulation exerts anti-chikungunya activity

**Figure 5:**
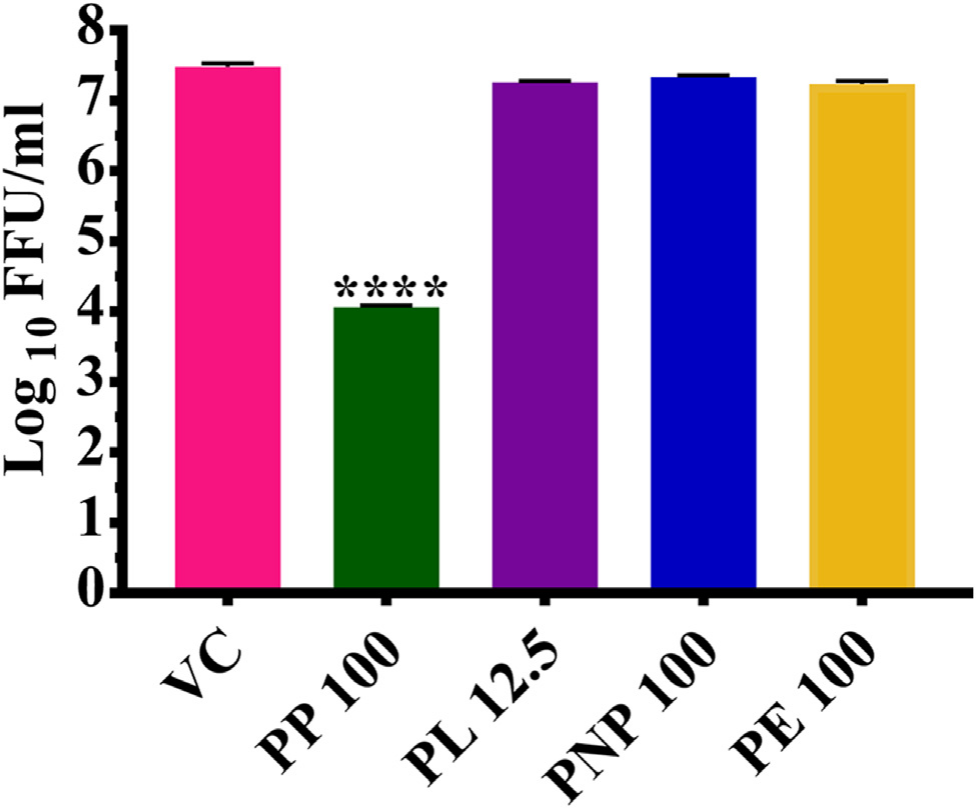
Primary screening of papaya formulations using FFU assay against CHIKV. Vero CCL-81 cells were infected with CHIKV for 1 h. and treated with the formulations. The cells infected were incubated for two days post infection. After incubation, plates were freezed at −80°C and thawed to collect culture supernatant which was used for the determination of titre of infectious virus particles by FFU assay. The results were expressed as mean log_10_FFU ± SEM. All the experiments were performed in triplicates. ∗∗∗∗p < 0.0001; ∗∗∗p < 0.001; ∗∗p < 0.01 and ∗p < 0.05 vs. virus control (VC).

**Figure 6:**
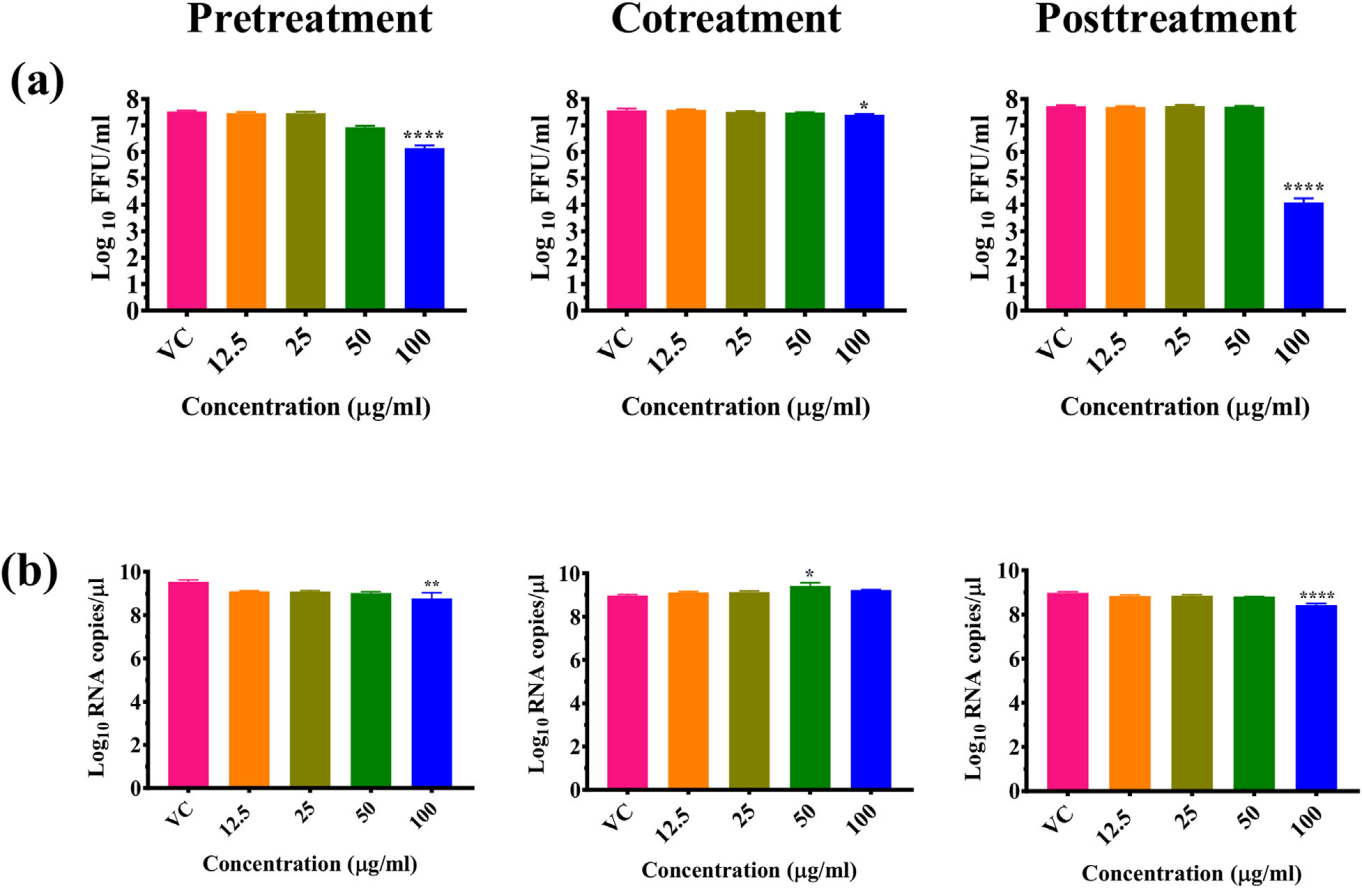
Effect of papaya powder on CHIKV under different treatment conditions by FFU assay (a) and qRT-PCR (b). Infected Vero CCL-81 cells were treated with various concentrations (12.5, 25, 50 and 100 μg/ml) of PNP and PE under different conditions. After incubation, the plates were freezed and the culture filtrates were used for FFU and qRT-PCR assays. Mouse anti-dengue antibody was used as a primary antibody and goat anti-mouse IgG HRP conjugate was used as a secondary antibody in the FFU assay. For qRT-PCR, viral RNA was isolated and used for estimating viral RNA copies. The results were expressed as mean log10RNA copies/μl or log10FFU/ml ± SEM. All the experiments were performed in triplicates at two different time points. ∗∗∗∗p < 0.0001; ∗∗p < 0.01 and ∗p < 0.05 vs. virus control (VC).

Primary screening of papaya based formulation for anti-chikungunya activity revealed that PP inhibited CHIKV (Figure 5). Further investigation of PP for anti-chikungunya activity under pretreatment, cotreatment and post infection treatment conditions revealed that the formulations inhibits CHIKV under posttreatment conditions at a concentration of 100 μg/ml from 7.73 (VC) to 4.08 log_10_ FFU/mL (p < 0.0001). In the case of pretreatment, a significant reduction of viral titre from 7.52 to 6.14 log_10_ FFU/mL (p < 0.0001) was noted while no effect was observed under the cotreatment conditions (Figure 6a). In the case of viral RNA copy number, no significant difference was observed (Figure 6b).

**Figure 7:**
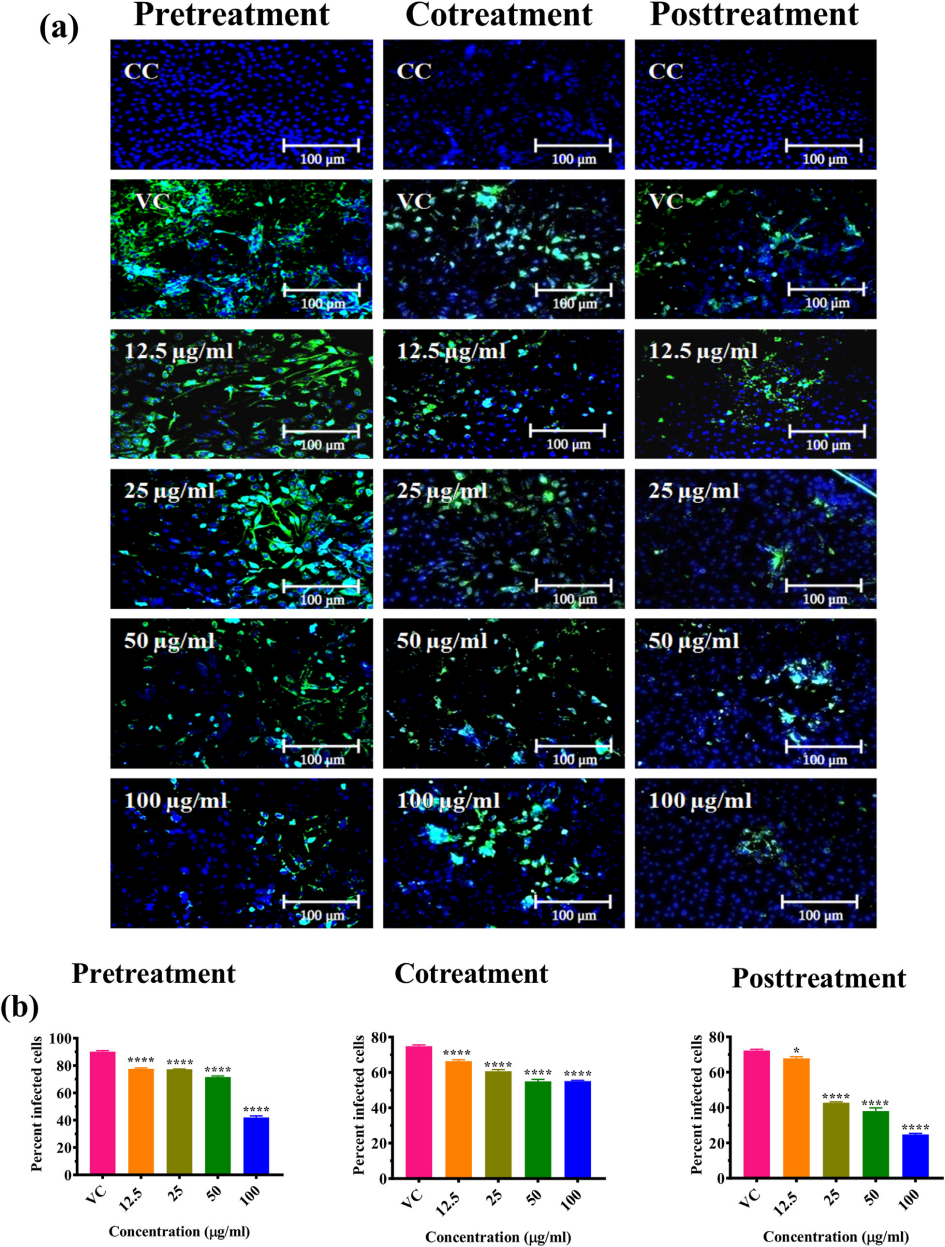
Effect of PP on CHIKV infection as measured by immunofluorescence assay. (a) Immunoflourescent images of Vero CCL-81 cells infected with CHIKV and treated with various concentrations of PP under different treatment conditions. Green colour cells indicate virus infected cells. (b) Percentage of infected Vero cells in cultures treated with various concentrations of PP under different conditions. Cells were counted in three different fields to obtain the percent infected cells. The results were expressed as mean percent-infected cells ± SEM. The experiments were performed in triplicates. ∗∗∗P < 0.001, ∗∗P < 0.01 and ∗P < 0.05 vs. virus control (VC).

IFA results demonstrated the dose dependent reduction of CHIKV antigen in cells treated with PP (Figure 7a and b) under all conditions. The highest reduction of infection was observed in cells treated with 100 μg/ml concentration of PP for CHIKV under post treatment conditions (Figure 7a and b).

### 3.2 Activity of commercially available *Carica papaya* products against DENV & CHIKV

The cytotoxicity of the drugs was determined by a methyl thiazolyl tetrazolium (MTT) assay on Vero CCL81 cells. MTT assay showed that Papayen, Tcyte and Platex were least cytotoxic with 50% cell toxicity (CC50) value > 250 μg/ml, followed by Platenza and Reditus with CC50 value of 48.04 μg/ml and 45.71 μg/ml respectively (Supplementary Figure 1).

Primary screening of commercially available papaya products for their anti-dengue and anti-chikungunya activity at MNTD revealed that there was no significant reduction in virus titre in drug treated cells compared to virus control (Supplementary Figure 2).

## 4 Discussion

Currently, no antiviral drugs are available for the treatment of dengue and chikungunya. Plant-derived antivirals act as a potential source for drug development. Papaya leaves are used as a natural cure for dengue by the public with much interest. Being easily available and affordable, the use of papaya leaves occurs indiscriminately. The efficacy of *Carica papaya* leaves formulation in drastically increasing platelet counts in dengue cases is one of the core topics of debate across the world. Although its role as an immunomodulator has been shown in some of the studies, there is no clear evidence for its use as an antiviral. In the present study, the anti-dengue activity of commercial papaya drugs as well four different formulations of PLE were investigated. Cytotoxicity analysis of the formulations revealed that except PL, all others were not cytotoxic. PL was not cytotoxic at lower concentrations. PL was prepared by heat treatment with water. It has been reported that heat treatment with water leads to extraction of more amount of total phenolics which might have contributed to the cytotoxicity observed with PL [23]. The study revealed among the four formulations, silver nanoparticles based on PLE (PNP) and Papaya extract prepared using a SF extraction machine (PE) showed activity against DENV -2 virus under all conditions though the effect was more pronounced in post infection treatment conditions. The results were confirmed by different assays such as FFU assay, which measures infectious virus titre, qRT-PCR that measures viral RNA copy number and IFA, which measures percent infected cells. However, results were not correlating exactly between the three assays; a trend was observed in all the assays. This could be because these assays measures different units. FFU assay measure only functional infectious virus particles while qRT-PCR assay measures both genomic RNA from mature and immature virus particles and replication intermediates. Since qRT-PCR is a highly sensitive assay and measure even very low quantity of RNA, the difference between the virus control and extract treated cultures might be reduced leading to low or loss of statistical significance. Though the antiviral activity was evaluated against only DENV-2, the formulations should be tested against the other serotypes. PNP was observed to be more potent than PE. The increased antiviral activity of PNP could be due to the presence of silver, which is a potent anti-microbial/viral agent. Though effect of silver nanoparticles in isolation has not been tested in this study, the increased inhibitory activity of PNP against DENV compared to other formulations suggests that addition of silver might have enhanced the activity, which needs further investigations. A recent study has shown that silver nanoparticles prepared from methanol based papaya extract had superior antiviral activity compared to crude PLE prepared using methanol as solvent [17]. Nanotechnology is being attempted extensively in drug delivery to improve the therapeutic outcomes of many diseases.

The commercial drugs with papaya as an active ingredient lack anti-dengue activity. The role of the other components of the drugs may influence the inhibitory activity. It is possible that these drugs might act as an immunomodulatory rather than an antiviral which needs exploration.

In previous studies, methanol extract of *Carica papaya* leaves and freeze dried CPLE didn't show any significant effect on DENV inhibition [24, 25]. The extraction of compounds depends on the solvent used. A single extraction method may not extract all the active compounds present in a plant and hence there is need to use multiple extraction methods. The extraction methods also certainly affect the pharmacological activity. For example, certain heat labile active compounds may be lost in Soxhlet extraction but remain intact in SFE methods. Extraction techniques also affect particle size. Extraction using a SF extraction machine is known to extract the active ingredients without affecting their properties while other extraction methods might affect the properties of active ingredients [6, 7, 26].

The SFE method is more accurate, safe, reliable, and fast with a higher yield and better-purified compounds. SFE leads to enhanced extraction efficiency with speed and selectivity due to its gas-like mass transfer properties and liquid-like solubility properties and environmental benefits. It can lead to extraction of analytes present in low concentrations, cleaner extracts, and preserve heat-labile bioactive constituents. It also produces extract with minimal organic solvent. Supercritical carbon dioxide is an excellent solvent for the extraction of non-polar, some low molecular weight, volatile, and polar compounds, but it is less effective in extracting phytochemicals present in the cell wall. SFE process can be enhanced by the addition of small amounts of organic co-solvent or solvent modifiers to increase the solvation power of scCO_2_ leading to better recovery of bioactive compounds.

The present study results revealed that *Carica papaya* powder, which is a crude extract of papaya leaves, exerted anti-chikungunya activity at a higher concentration under all conditions. However, this anti-chikungunya activity was not observed in other papaya formulations used in the present study. PL which was prepared as PP except for heat treatment with water showed cytotoxicity and hence, a lower concentration of PL used in antiviral study which could be the reason for its lack of anti CHIKV activity. Concentration of the active compound might be less than the threshold concentration required to exert anti-chikungunya activity in other formulations. Whether extraction methods had affected the anti-chikungunya activity needs further investigations. This is the first study to report anti-chikungunya activity of CPLE.

Identifying the active components exerting anti-dengue and anti-chikungunya activities will help in developing new drugs. Quercetin, a bioflavanoid, present in papaya has been shown to be effective against DENV [27]. In silico studies have shown the interaction of quercetin with dengue NS2B-NS3 protease [28]. Quercetin has been reported to inhibit O'nyong-nyong virus, a virus related to CHIKV, on co treatment in human synovial fibroblast cells [29]. The effect of Quercetin also needs to be investigated against CHIKV. The main limitation of this preliminary study is that the chemical composition and the bioactive molecules in the PLE were not determined. Identification of the active component from PLE which are active against both DENV and CHIKV and synthesis and evaluation of such drugs may be considered in long term. However, developing methods to prepare inexpensive papaya formulations which preserve the active component with anti-dengue and anti-chikungunya activity without lot to lot variations and further preclinical studies in animal models and clinical trials using those herbal formulations is warranted immediately.

## 5 Conclusion

The present study concludes that CPLE formulations exhibit anti-dengue and anti-chikungunya properties and needs further exploration to identify the bioactive compounds.

## Declarations

### Author contribution statement

Poonam Patil: Performed the experiments; Analyzed and interpreted the data.

Kalichamy Alagarasu; Sarah Cherian; Deepti Parashar: Conceived and designed the experiments; Analyzed and interpreted the data; Wrote the paper.

Deepika Chowdhury: Performed the experiments; Analyzed and interpreted the data; Wrote the paper.

Mahadeo Kakade: Performed the experiments.

Sulochana Kaushik; JP Yadav: Contributed reagents, materials, analysis tools or data.

Samander Kaushik: Contributed reagents, materials, analysis tools or data; Wrote the paper.

### Funding statement

This work was supported by ICMR-National Institute of Virology, Pune, India.

### Data availability statement

Data included in article/supp. material/referenced in article.

### Declaration of interest's statement

The authors declare no conflict of interest.

### Additional information

No additional information is available for this paper.

## Appendix A Supplementary data

Supplementary Figure 1.tiff Effect of commercially available papaya based drugs on the cell viability of Vero CCL81 cells using the MTT assay. Vero CCL81 cells were incubated with various concentrations of drugs for 24 h. The results were obtained at 570nm absorbance and shown as percentages cell viability. Supplementary Figure 1.tiff

Supplementary Figure 2.tiff Primary screening of commercially available drugs using Foci forming unit reduction assay against DENV (a) & CHIKV (b). Vero CCL81 cells were infected with DENV & CHIKV with a multiplicity of infection of 0.1 and 0.01 respectively. Four hours post infection cells were treated with drugs at their maximum non-toxic doses -250 μg/ml for Papayen, Tcyte, and Platex and 7.8 μg/ml Reditus and plaza respectively. Five days post-infection cells were subjected to FFU assay. Log 10 virus titre in drug-treated cells was compared to virus control (VC). Supplementary Figure 2.tiff

Supplementary Table 1_V2.docx Supplementary Table 1_V2.docx
