# Supplementary figures and images for: In-vitro antiviral activity of Carica papaya formulations against dengue virus type 2 and chikungunya virus
Source: Heliyon. 2022 Nov 30;8(12):e11879. doi: 10.1016/j.heliyon.2022.e11879 (PMC9723942; doi:10.1016/j.heliyon.2022.e11879)

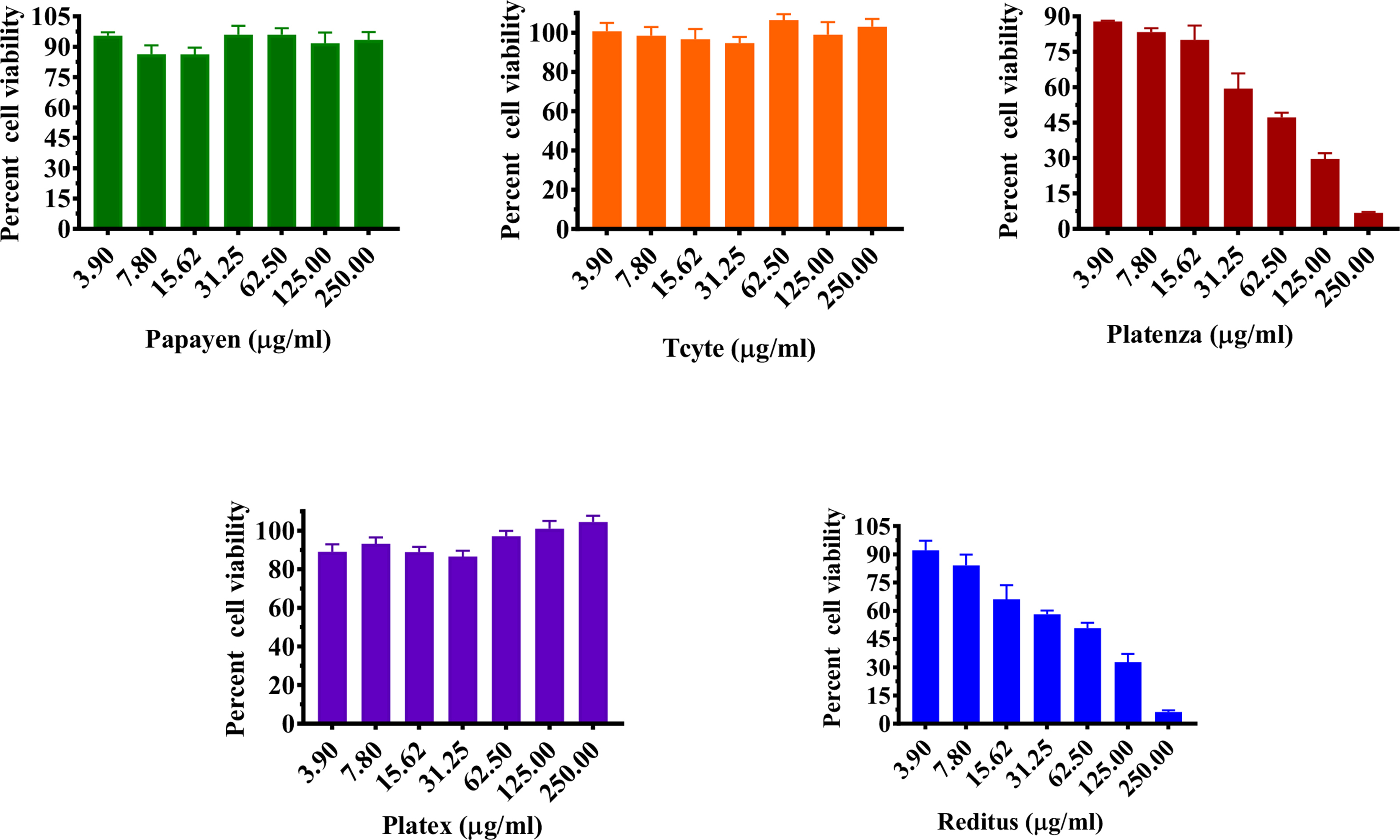

Supplement: Supplementary Figure 1.tiff — Effect of commercially available papaya based drugs on the cell viability of Vero CCL81 cells using the MTT assay. Vero CCL81 cells were incubated with various concentrations of drugs for 24 h. The results were obtained at 570nm absorbance and shown as percentages cell viability. [file figs1.jpg]

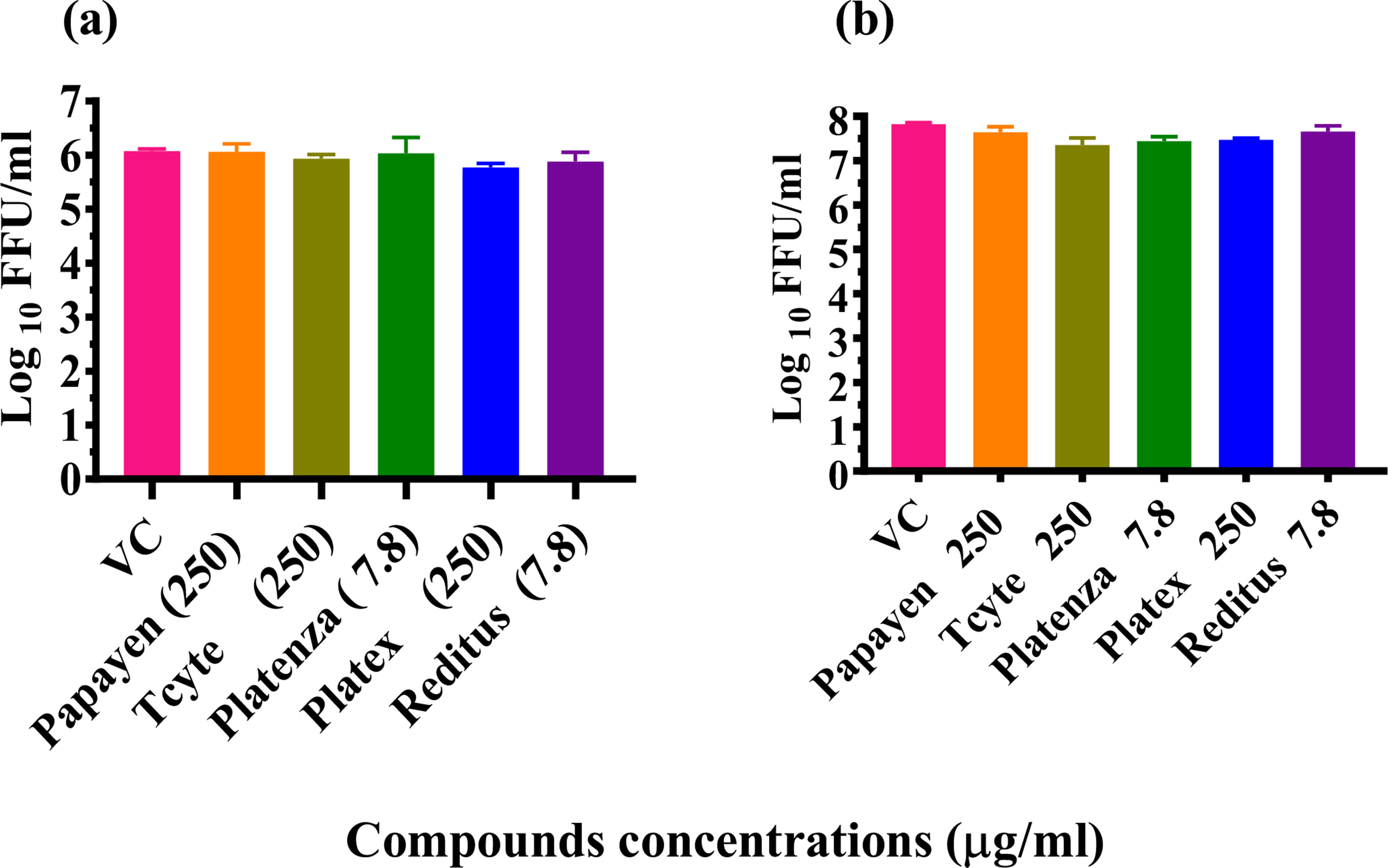

Supplement: Supplementary Figure 2.tiff — Primary screening of commercially available drugs using Foci forming unit reduction assay against DENV (a) & CHIKV (b). Vero CCL81 cells were infected with DENV & CHIKV with a multiplicity of infection of 0.1 and 0.01 respectively. Four hours post infection cells were treated with drugs at their maximum non-toxic doses -250 μg/ml for Papayen, Tcyte, and Platex and 7.8 μg/ml Reditus and plaza respectively. Five days post-infection cells were subjected to FFU assay. Log 10 virus titre in drug-treated cells was compared to virus control (VC). [file figs2.jpg]
