# Supplementary material for: In-vitro antiviral activity of Carica papaya formulations against dengue virus type 2 and chikungunya virus
Source: Heliyon. 2022 Nov 30;8(12):e11879. doi: 10.1016/j.heliyon.2022.e11879 (PMC9723942; doi:10.1016/j.heliyon.2022.e11879)
Supplement: Supplementary Table 1_V2.docx [file mmc1.docx]

| S. No. | Commercial product | Ingredients | Quantity |
| --- | --- | --- | --- |
| 1. | Tcyte  (Each 5 ml contains) | Ext. Papaya  Ext. Aloe vera  Ext. Madhukparni  Ext. Anwla  Ext. Haritaki  Ext. Gilloy  Ext. Danamethi  Ext. Punarnava  Ext. Tulsi  Ext. Tikakatu | 1200 mg  200 mg  400 mg  200 mg  100 mg  100 mg  100 mg  100 mg  100 mg  100 mg |
| 2. | Papayen  (Each 5 ml contains) | *Carica papaya*  Dhataki Pushpa (*Woodfodia fruticosa*)  Sugar | 2500 mg  150 mg  1400 mg |
| 3. | Reditus  (Each capsule contains) | *Carica papaya*  *Tinospora cordiofolia*  *Emblica officinalis*  *Ocimum sanctum*  *Sida cordiofolia*  *Punica granatum*  *Allium sativaum*  Wheat grass | 150 mg  75 mg  75 mg  50 mg  50 mg  25 mg  25 mg  25 mg |
| 4. | Platex  (Each capsule contains) | Papaya leaf extract  Guduchi stem extract  Vasa leaf extract  Pippali fruit extract | 150 mg  100 mg  100 mg  10 mg |
| 5. | Platenza  (Each capsule contains) | *Carica papaya*  *Phyllanthus maderaspatensis*  Guduchi  Maricha (*Piper nigrum*) | 265.7 mg  95.6 mg  35.4 mg  3.3 mg |

Supplementary Table 1: Composition of commercially available drugs containing papaya as an active ingredient
